# A-Kinase Anchoring in Dendritic Cells Is Required for Antigen Presentation

**DOI:** 10.1371/journal.pone.0004807

**Published:** 2009-03-11

**Authors:** Robynn V. Schillace, Casey L. Miller, Neal Pisenti, Jeff E. Grotzke, Gwendolyn M. Swarbrick, David M. Lewinsohn, Daniel W. Carr

**Affiliations:** 1 Portland Veterans Affairs Medical Center, Oregon Health and Science University, Portland, Oregon, United States of America; 2 Department of Neurology, Oregon Health and Science University, Portland, Oregon, United States of America; 3 Department of Microbiology and Molecular Immunology, Oregon Health and Science University, Portland, Oregon, United States of America; 4 Department of Pulmonary Medicine, Oregon Health and Science University, Portland, Oregon, United States of America; 5 Department of Endocrinology, Oregon Health and Science University, Portland, Oregon, United States of America; New York University School of Medicine, United States of America

## Abstract

**Background:**

Dendritic cells (DC) are the most potent antigen presenting cells (APC) of the immune system. Prostaglandin E_2_, cyclic AMP, and protein kinase A (PKA) have all been shown to regulate DC maturation and activity. In other cells, the ability of these molecules to convey their signals has been shown to be dependent on A-kinase anchoring proteins (AKAPs). Here we present evidence for the existence and functional importance of AKAPs in human DC.

**Methodology/Principal Findings:**

Using immunofluorescence and/or western analyses we identify AKAP79, AKAP149, AKAP95, AKAP LBC and Ezrin. We also demonstrate by western analysis that expression of AKAP79, AKAP149 and RII are upregulated with DC differentiation and maturation. We establish the functional importance of PKA anchoring in multiple aspects of DC biology using the anchoring inhibitor peptides Ht31 and AKAP-IS. Incubation of protein or peptide antigen loaded DC with Ht31 or AKAP-IS results in a 30–50% decrease in antigen presentation as measured by IFN-γ production from antigen specific CD4^+^ T cells. Incubation of LPS treated DC with Ht31 results in 80% inhibition of TNF-α and IL-10 production. Ht31 slightly decreases the expression of CD18 and CD11a and CD11b, slightly increases the basal expression of CD83, dramatically decreases the LPS stimulated expression of CD40, CD80 and CD83, and significantly increases the expression of the chemokine receptor CCR7.

**Conclusions:**

These experiments represent the first evidence for the functional importance of PKA anchoring in multiple aspects of DC biology.

## Introduction

Dendritic cells (DC) are professional antigen presenting cells (APC) capable of stimulating resting T cells to generate an antigen specific primary immune response. DC capture, process and present antigen bound to MHC for recognition by and subsequent activation of T cells [1].

Recent studies indicate that prostaglandin E_2_ (PGE_2_) is produced by DC upon maturation [2]. PGE_2_ can stimulate production of the small molecule second messenger cyclic AMP (cAMP). In murine DC, cAMP can inhibit antigen presentation, MHC class II expression and tumor necrosis factor-α (TNF-α) production, and can increase interleukin-10 (IL-10) production, resulting in overall suppression of the immune response [3]. In contrast to the murine system, cyclic AMP elevating agents have been shown to stimulate human DC inducing their activation and migration [4], [5]. One mediator of cAMP action is protein kinase A (PKA). PKA is a serine/threonine protein kinase that is involved in promoting DC maturation and inhibiting IL-12 and TNF-α production [6], [7]. PKA is a holoenzyme comprised of a dimer of regulatory subunits each of which binds one catalytic subunit. There are four isoforms of regulatory subunits (RIα, RIβ and RIIα, RIIβ), and three isoforms of catalytic subunits (Cα, Cβ, Cγ). The catalytic subunits are inactive when bound to the regulatory subunits and are activated by cAMP binding to the regulatory subunits inducing the release and activation of the catalytic subunits [8].

It is now widely accepted that PKA actions are regulated spatially and temporally through interactions of the regulatory subunits with A-kinase anchoring proteins (AKAPs). AKAPs are a family of proteins that mediate the effects of cAMP by targeting two effectors of cAMP, PKA and the exchange protein directly activated by cAMP (Epac) [9], [10]. Disruption of PKA binding to AKAPs in mouse and human T cells blocks the ability of cAMP to inhibit T cell activation [11], [12]. However, the involvement or even the presence of AKAPs in dendritic cells has not yet been reported. Here we present the novel findings that AKAPs are present in dendritic cells and that AKAP expression is differentially induced upon maturation from a monocyte to a mature DC. Additionally, we demonstrate that type II PKA binding to AKAPs is necessary for optimal antigen presentation and activation of CD4^+^ T cells, for TNF-α and IL-10 production, and for cell surface expression of costimulatory molecules, integrins, and the chemokine receptor CCR7.

## Results

### AKAPs are expressed in dendritic cells

**Figure 1:**
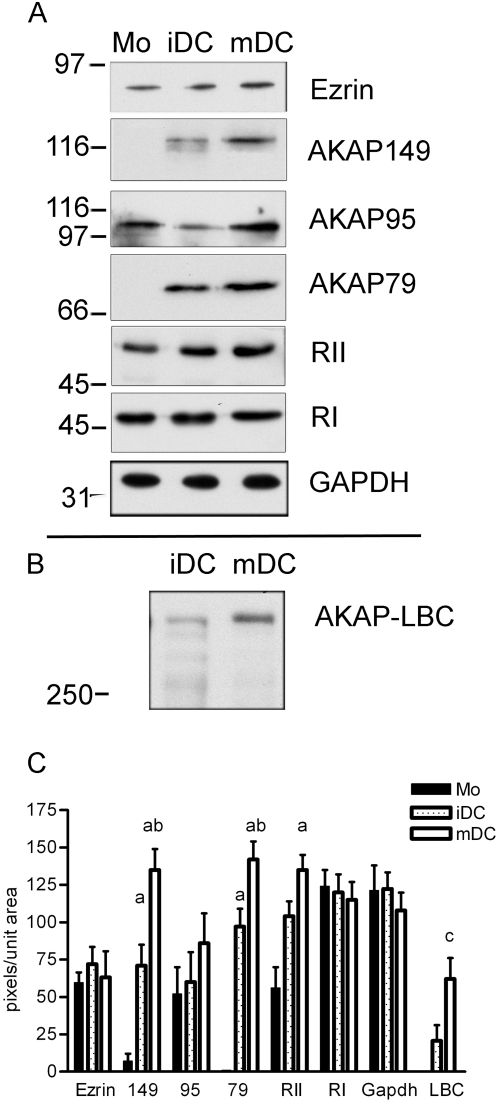
Identification of AKAPs in dendritic cells by western analysis. Monocytes were isolated from PBMC by negative isolation of CD14^+^ cells (Mo), plated in culture for 5 days with GM-CSF and IL-4 (immature DC; iDC) or plated for five days with GM-CSF and IL-4 and an additional two days with LPS (mature DC; mDC). See Materials and Methods for details. A) One representative set of westerns in which 10 µg total protein was loaded in each lane. Membranes were probed with monoclonal antibodies to AKAP149, AKAP95, AKAP79, RIIα, RIα, and GAPDH, goat-anti-mouse HRP conjugated secondary and chemiluminescence detection. B) One representative western in which 10 µg of total protein was loaded in each lane. The membrane was probed with a rabbit polyclonal antibody to AKAP-Lbc (generous gift of Dr. Diviani), goat-anti-rabbit HRP conjugated secondary and chemiluminescence detection. C) Quantitation of five westerns using NIH image quantitation software. One way ANOVA for three correlated samples yielded significance with p = 0.0004 for AKAP149; p<0.0001 for AKAP79, and p = 0.003 for RIIα. A Tukey HSD test confirmed statistical significance between Mo and iDC (a) and between iDC and mDC (b) p<0.05. Quantitation of three westerns and student ttest for AKAP-Lbc yielded statistical significance (c) p = 0.005.

The ability of a dendritic cell to process and present antigen and to stimulate T cells varies with maturation state [13]. Thus, we examined the expression of AKAPs in CD14 purified monocytes (MO), immature dendritic cells (iDC; 5 days with GM-CSF and IL-4) and mature dendritic cells (mDC; two additional days with LPS) by western analyses. As depicted in Figure 1, Ezrin, AKAP79, AKAP149, AKAP95, and AKAP LBC were detected. Well characterized antibodies for Ezrin, AKAP79, AKAP149, AKAP95, RIIα, RIα, and GAPDH recognize a single band of the expected molecular weight as indicated. AKAP-Lbc antibody recognized several bands of lower molecular weights, which may be break down products of the full length protein. Figure 1A is one representative set of westerns. Quantitation of five sets of westerns for Ezrin, AKAP79, AKAP149, AKAP95, RIIα, RIα, and GAPDH illustrated that the expression of AKAP149 and AKAP79 was upregulated 10 and 100 fold, respectively, as the monocytes became immature DC (iDC) and increased again 1.9 and 1.5 fold as the immature DC fully matured (mDC) (Figure 1C). AKAP 95 and Ezrin were detected in relatively equal amounts at each stage as any differences are not statistically significant. The amount of the RIIα regulatory subunit of PKA expressed increased 1.8 fold as the monocyte became an immature DC, however, the additional increase in RIIα signal between the immature and mature DC was not statistically significant. The RIα regulatory subunit of PKA was detected at equal levels in each cell population. GADPH was used as a loading control and shows no significant changes in expression among the three cell populations. AKAP-Lbc was detected in iDC and mDC with a significant (3 fold) upregulation of AKAP-Lbc between iDC and mDC (Figure 1B). Three sets of AKAP-Lbc westerns were quantitated. AKAP-Lbc protein was only detected when cells were lysed in Lbc lysis buffer (see materials and methods). Protein quality from monocytes was not acceptable using this method of cell lysis; therefore we make no claims about the presence of AKAP-Lbc in monocytes. These data identify specific AKAPs in DC and further illustrate that the expression of some AKAP and PKA proteins are upregulated with DC maturation.

### Subcellular localization of AKAPs

**Figure 2:**
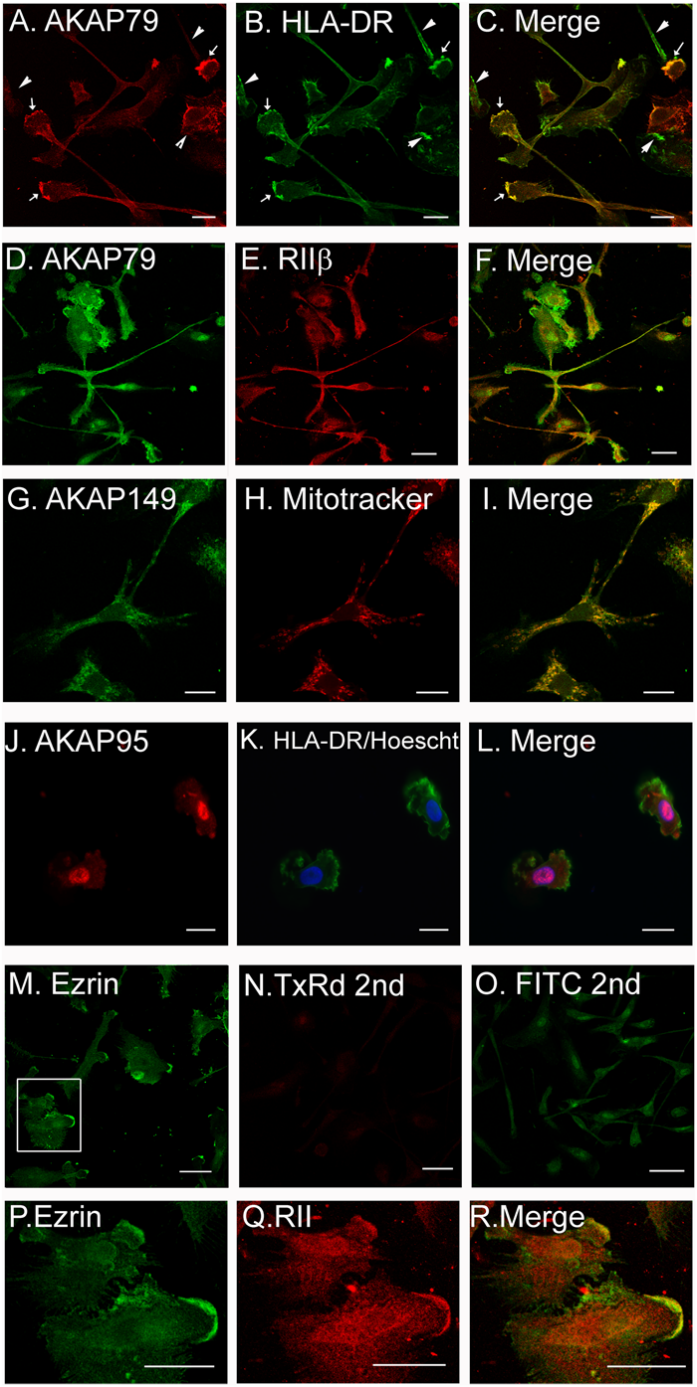
Immunofluorescence of AKAPs in dendritic cells. Monocytes derived from adherence to plastic were plated onto poly-l-lysine coated glass coverslips. The cells were incubated for 30 minutes with mitotracker (panels H, I), and/or fixed, permeabilized, and stained with monoclonal antibodies to AKAP79 (panels A, C, D, F), AKAP149 (panels G, I), AKAP95 (panels J, L), HLA-DR (panels B, C, K, L), Ezrin (panels M, P), RIIβ (panels, E, F, Q, R). Hoescht (panel K, L) was added with secondary antibody conjugates. Panels P-R are 2 fold zoom of the boxed area of panel M. Scale bar = 25 µm.

The ability of AKAPs to coordinate cAMP signaling events is through spatio-temporal localization of signaling molecules bound to the AKAP. Localization is mediated by unique targeting domains within each AKAP. Using immunofluorescent confocal microscopy we illustrate that AKAP79, AKAP149, AKAP95, and Ezrin are detected in unique subcellular locations in mature DC. Antibody to HLA-DR was used to delineate the cell (Figure 2 panels B, C, K, and L). AKAP79 was detected in the membrane (Figure 2; panels A, C, D, and F). In some cells AKAP79 staining overlaps strikingly with HLA-DR staining (panels A–C, arrows); while in other cells staining for AKAP79 and HLA-DR appears to be exclusive (panels A–C, arrowheads). As expected, AKAP79 staining also overlaps with RII staining (Figure 2; panels D–F). AKAP149 was detected predominantly in the mitochondria with some nuclear staining (Figure 2; panels G–I). Mitochondrial staining was confirmed by overlapping signals with the mitochondrial marker mitotracker. Staining for AKAP95 reveals a characteristic pattern of nuclear expression, HLA-DR was used to provide a cellular outline and hoescht stain was used to confirm nuclear localization (Figure 2; panels J–L). Ezrin staining is strongest in the membrane ruffles and leading edge of the cells (Figure 2; panel M). A zoom of the outlined region in panel M is used to illustrate overlapping Ezrin and RII staining in the membrane (Figure 2; panels P–R). Secondary antibody staining for Texas Red conjugates is minimal (Figure 2; panel N). Secondary antibody staining for FITC conjugates is significant but different than most of the primary staining of interest (Figure 2; panel O). The majority of the FITC secondary staining was in the nucleus, thus this conjugate was not used for AKAP95 visualization. The pattern of staining in immature DC was found to be similar to the mature DC (data not shown) suggesting that the changes present in protein expression (Figure 1) are likely in the amount of protein being produced rather than changes in localization.

### Disrupting PKA anchoring inhibits antigen presentation

**Figure 3:**
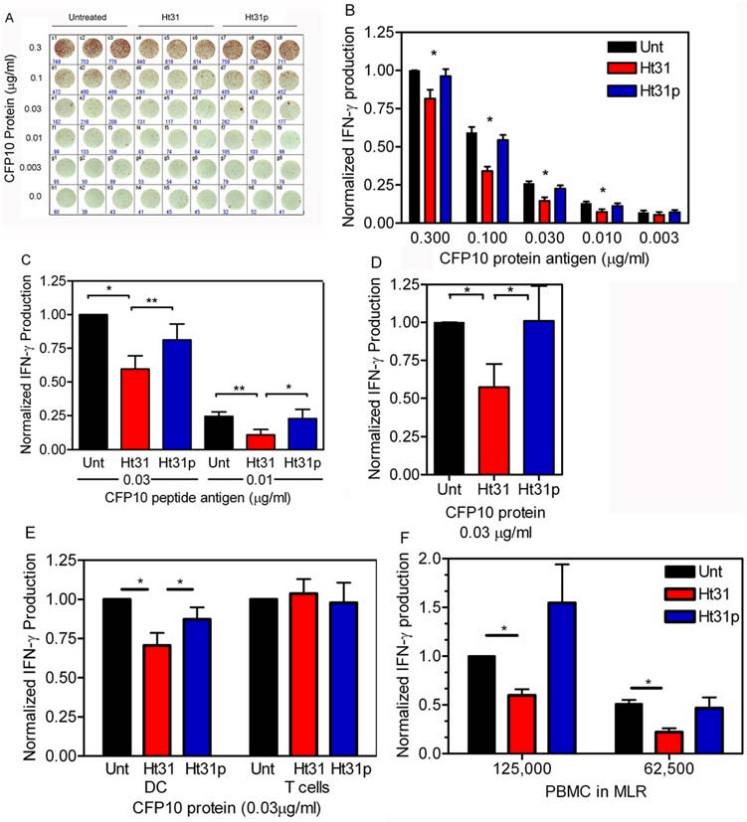
Anchored PKA is required for antigen presentation as assayed by IFN-γ ELISPOT. A) One representative ELISPOT. Day 5 dendritic cells (iDC) were incubated without (Unt, black bars) or with Ht31 (red bars) or Ht31p (blue bars), and the indicated concentration of CFP10 protein (B) or CFP10 peptide antigen (C) for 1 hr at 37°C. Antigen specific CD4^+^ clonal T cells were added and IFN-γ production was assayed after 16 hours by ELISPOT. D) Allogeneic day 5 dendritic cells (iDC) were incubated without (Unt, black bars) or with Ht31 (red bars) or Ht31p (blue bars) for 1 hr at 37°C, 5% CO_2_. The cells were collected, rinsed and replated. Then 0.03 µg/ml of protein antigen (CFP10) was added for 1 hr at 37°C, 5% CO_2_, and antigen specific CD4^+^ clonal T cells were added. IFN-γ production was assayed after 16 hours by ELISPOT. E) First set of three bars, experiment is conducted as in (B). Second set of three bars, antigen specific CD4^+^ clonal T cells were incubated without (Unt, black bars) or with Ht31 (red bars) or Ht31p (blue bars) for 1 hr at 37°C, rinsed, and added to allogeneic day 5 DC preloaded with CFP10 protein antigen (0.03 µg/ml). IFN-γ production was assayed after 16 hours by ELISPOT. The data presented are an average of three independent experiments with values (number of spots counted) normalized to the response at 0.03 µg/ml CFP10 protein (B), 0.03 µg/ml CFP10 peptide (C). F) MLR, CD14^+^ monocytes were matured and 10,000 DC were plated with 125,000 or 62,500 PBMC as indicated, without (Unt, black bars) or with 100 µM Ht31 (red bars) or Ht31p (blue bars). In all figures error bars represent S.E.M. (B) Two factor ANOVA with repeated Measures on Both Factors was conducted on the un-normalized values (actual count of number of cells making IFN-γ, P<0.05). (C, D, E, F) A student TTEST yielded significance with a P<0.05 (*) or P<0.01 (**).

To determine if AKAPs have a function in regulating antigen presentation, we disrupted the PKA/AKAP interaction using the Ht31anchoring inhibitor and Ht31p control peptides. The anchoring inhibitor Ht31 is a peptide that forms an amphipathic helix which binds to RII preventing its binding to AKAPs. The control peptide, Ht31p, incorporates a proline mutation which kinks the helix, thwarting its ability to bind and disrupt PKA anchoring. Immature DC (day 5) were treated with Ht31 or the Ht31p control peptide and loaded with either CFP10 protein or peptide antigen at the indicated concentrations. CFP10 is an immunodominant Tuberculosis antigen used here as a tool to observe antigen presentation and T cell activation. D454 E12 clonal CD4^+^ T cells specific for CFP10 were then added to the DC. T cell activation was measured by ELISPOT analysis of IFN-γ production (see material and methods). The ability of the DC to present antigen and activate the T cells was significantly reduced in the presence of the Ht31 peptide. Figure 3A is one representative ELISPOT of an antigen titration experiment. The experiment was repeated at least three times using DC from different matched and unmatched donors. The data were normalized and averaged for graphical presentation in Figure 3B. IFN-γ production (spot forming units) was reduced an average of 33% when comparing Ht31 treated DC to untreated DC and 28% when comparing Ht31 treated DC to Ht31p treated DC, in CFP10 protein loaded cells. When CFP10 peptide was used as antigen, IFNγ production was reduced 50% in Ht31 treated DC versus untreated DC. The reduction was 39% when comparing Ht31 treated DC to Ht31p treated DC (Figure 3C). These data suggest that PKA anchoring is required for optimal antigen presentation and T cell activation.

To confirm that Ht31 is disrupting PKA anchoring in the dendritic cells and not in the T cells, two different experiments are presented. Ht31 or Ht31p were added to day 5 immature DC with 0.03 µg/ml CFP10 protein antigen. The cells were incubated for 1 hour at 37°C and rinsed to remove the peptides. Fresh media and the D454 E12 clonal T cells were then added and IFN-γ production was assayed by ELISPOT. As presented in Figure 3D, Ht31 added to the DC inhibited the ability of the DC to activate the T cells. IFN-γ production from the T cells in Ht31 treated DC was reduced 43% compared to untreated control DC and Ht31p treated DC, suggesting that Ht31 is disrupting PKA anchoring and antigen presentation within the DC. In Figure 3E, D454 E12 clonal CD4^+^ T cells were treated with 100 µM Ht31 or Ht31p for 1 hour at 37°C, the T cells were rinsed and added to antigen loaded DC and IFN-γ production was assayed by ELISPOT. Consistent with figure 3b, the first set of bars illustrate, IFN-γ production is inhibited in Ht31 treated DC. However, the second set of bars reveals that when the T cells are treated with Ht31, IFN-γ production is not inhibited. These data provide evidence that the inhibition of IFN-γ production presented in these experiments is the result of Ht31 disrupting anchoring within the DC.

To illustrate that inhibition of T cell activation is not specific to T cell clones, we performed a mixed lymphocyte reaction (MLR). In Figure 3F, day 5 DC matured from CD14^+^ monocytes were incubated without or with 100 µM Ht31or Ht31p and PBMC for 3 days. IFN-γ production was measured by ELISPOT and presented as normalized and averaged cytokine activity. The data illustrate that Ht31 treatment of the DC significantly inhibits T cell activation 40–56% depending on the number of PBMC used (40% using 125,000 PBMC, 56% using 62,500 PBMC). The control Ht31p peptide does not inhibit T cell activation. These data confirm that the ability of Ht31 to disrupt antigen presentation and T cell activation is not specific to T cell clones and can be illustrated in peripheral blood T cells. Collectively these data demonstrate that PKA anchoring is important in the regulation of antigen presentation and T cell activation by DC.

**Figure 4:**
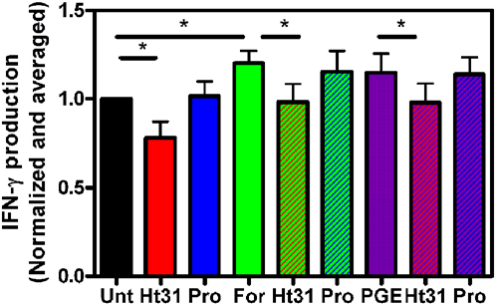
Ht31 blocks the ability of PKA activators to stimulate antigen presentation and T cell activation. Day 5 DC were incubated in the absence (black bar) or presence of 10 µM forskolin (green bars) or 1 µM PGE_2_ (purple bars) and in the absence or presence of 100 µM Ht31 (red and red hatched bars) or 100 µM Ht31p (blue and blue hatched bars). CFP10 protein antigen (0.03 µg/ml) was added and after 1 hr incubation at 37°C, 5% CO_2_, antigen specific CD4^+^ clonal T cells were added. IFN-γ production was assayed by ELISPOT after 16 hr at 37°C, 5% CO_2_. Six experiments were normalized and averaged. Error bars are S.E.M. Student TTEST reveals significance P<0.05(*).

To examine the ability of Ht31 to reverse the effects of known PKA activators we performed ELISPOT analyses in the presence of forskolin and PGE_2_. In agreement with published studies using human DC, forskolin (10 µM) and PGE_2_ (1 µM) increased antigen presentation (Figure 4, green and purple bars, respectively). Ht31 reversed the effects of forskolin and PGE_2_ (Figure 4, green-red hatched bars and purple-red hatched bars), respectively while Ht31pro did not (Figure 4, green-blue hatched and purple-blue hatched bars, respectively). These results support the hypothesis that Ht31 inhibits antigen presentation by interfering with PKA signaling.

**Figure 5:**
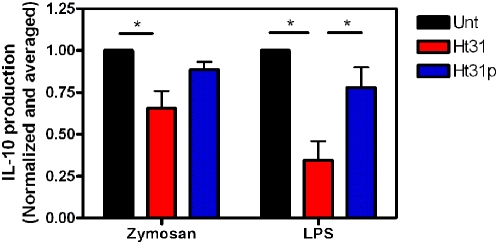
Ht31 inhibits secretion of IL-10. Day 5 DC were incubated with 30 µg/ml Zymosan or 10 ng/ml LPS, without (Unt, black bars) or with 100 µM Ht31 (red bars) or Ht31p (blue bars) for 24 hours at 37°C, 5% CO_2_. Supernatants were collected and used in IL-10 ELISA (R&D Systems). Three experiments were normalized and averaged. Error bars are S.E.M. Student TTEST reveals significance, P<0.05(*) or P<0.005(**).

We next examined the ability of DC to secrete the immunosuppressive cytokine IL-10 in the presence of Ht31 or Ht31p when stimulated with Zymosan or LPS. Day 5 DC were incubated with 30 µg/ml Zymosan or 10 ng/ml LPS overnight and supernatants were used in ELISAs. The data illustrate that 100 µM Ht31 inhibited the secretion of IL-10 from Zymosan treated cells by 35% and LPS treated cells by 65% (Figure 5, red bars). The control peptide Ht31p has no significant effect on cytokine secretion. These data suggest that PKA signaling is important for IL10 secretion.

**Figure 6:**
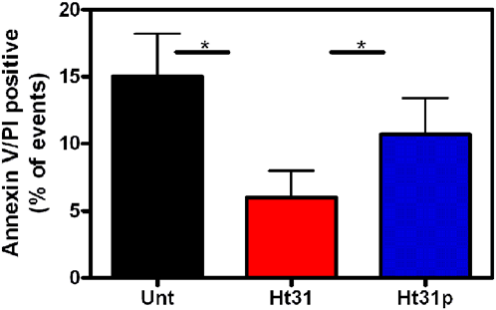
Ht31 is not inducing cell death. Day 5 DC were incubated without (Unt, black bars) or with 100 µM Ht31 (red bars) or Ht31p (blue bars) for 48 hours, collected, stained with annexin V and propidium iodide and analyzed by FACS. Percent annexin V/PI positive events from three different donors were averaged. Error bars are S.E.M. Student ttest reveals significance, P<0.05(*).

The robust inhibition IL-10 secretion prompted examination of cell viability in the presence of Ht31 and Ht31p. Day 5 DC were treated with Ht31 or Ht31p overnight and stained with annexin V/PI. An average of three experiments illustrates that less than 20% of DC exhibited annexin V/PI positive staining (Figure 6, black bar). Incubation with Ht31 reduced the annexin V/PI staining to 6% (Figure 6, red bar), while Ht31p treated cells averaged 10.5% annexin V/PI positive staining (Figure 6, blue bar). These differences were found to be statistically significant (P<0.05) indicating that Ht31 is not killing the cells and may in fact be preventing cell death.

**Figure 7:**
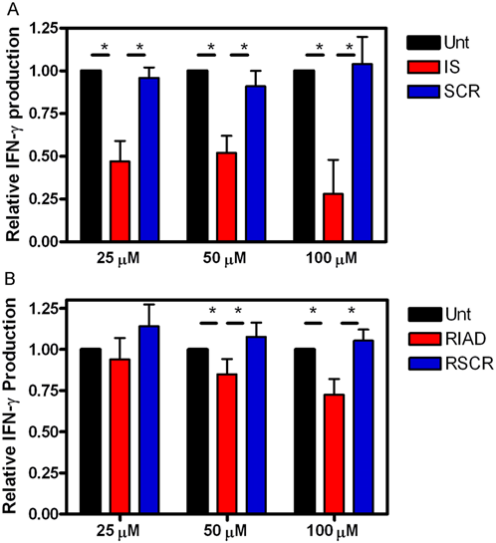
Inhibition of antigen stimulation is mediated by signaling from type II PKA. Allogeneic day 5 dendritic cells (iDC) were incubated without (Unt, black bars) or with A) the indicated concentrations of type II anchoring inhibitor (IS, red bars) or the control (SCR, blue bars), or B) the indicated concentrations of type I anchoring inhibitor (RIAD, red bars) or the control (RSCR, blue bars) for 1 hr at 37°C, 5% CO_2_. The cells were collected, rinsed and replated. Then 0.1 µg/ml of protein antigen (CFP10) was added for 1 hr at 37°C, 5% CO_2_, and antigen specific CD4^+^ clonal T cells were added. IFN-γ production was assayed after 16 hours by ELISPOT. Data are an average of three donors. Error bars are S.E.M. Student ttest reveals significance, P<0.05(*).

To dissect which PKA isoforms were contributing to the inhibition of antigen presentation we performed ELISPOT experiments using the RII selective inhibitor AKAP-IS and the RI selective inhibitor RIAD along with their control peptides SCR and RSCR [14], [15]. The poly-arginine containing peptides were added to DC at the indicated concentrations, 0.1 µg/ml CFP10 protein antigen and D454 E12 CD4^+^ clonal T cells were added as in figure 3a. Interferon-γ production from three experiments was normalized and averaged and is presented in figure 7A. Antigen presentation is inhibited approximately 50% by 25 µM AKAP IS. Inhibition increases to about 75% with 100 µM AKAP IS. In contrast, RIAD does not significantly inhibit antigen presentation at 25 µM and inhibits about 25% at 100 µM (Figure 7B). Studies in T cells suggest that RIAD should disrupt type I PKA anchoring at concentrations less than 10 µM [15]. Thus these data suggest that antigen presentation is more dependent on signaling via the RII isoforms of PKA.

**Figure 8:**
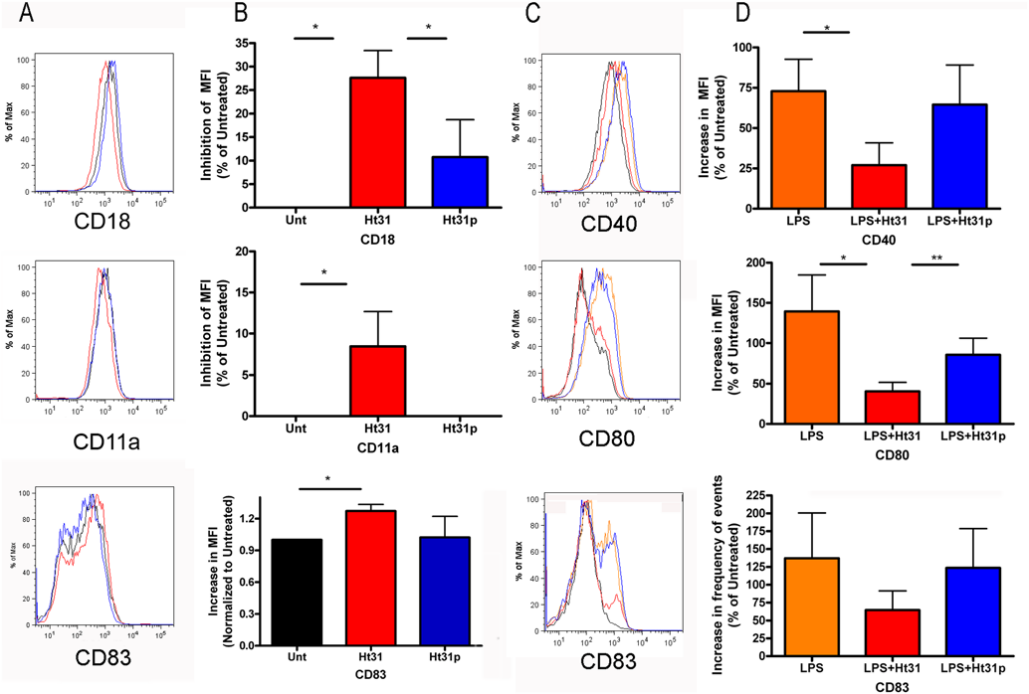
Ht31 inhibits cell surface expression of CD18, CD40, CD80 and CD83. Day 5 DC were incubated without or with 10 ng/ml LPS and 100 µM Ht31 or Ht31p for 48 hours. Cells were collected, blocked, and stained for cell surface expression using conjugated antibodies, see materials and methods. FACS analysis was performed on a LSRII using FacsDiva software. A) One representative histogram showing untreated cells (black trace), Ht31 treated cells (red trace), and Ht31p treated cells (blue trace). B) Graphical representation of percent inhibition of mean fluorescent intensities compared to untreated samples, data from eight donors was normalized and averaged. C) One representative histogram showing untreated cells (black trace), LPS treated cells (orange trace), LPS+Ht31 (red trace), and LPS+Ht31p (blue trace). D) Graphical representation of percent increase in mean fluorescent intensity compared to untreated cells, data from five donors was normalized and averaged. Error bars are S.E.M. Student ttest yielded statistical significance as indicated, P<0.05 (*), P<0.1 (**).

To determine if the suboptimal stimulation of T cells by DC was due to regulation of costimulatory molecules or integrins, the expression of CD18, CD11a, CD11b, CD11c, CD40, CD80, CD83, CD49d, CD86, and HLA-DR on DC were examined. Day 5 DC were incubated without or with Ht31 or Ht31p, and without or with Ht31 or Ht31p in the presence of LPS for 48 hours at 37°C, 5% CO_2_. In figure 8 columns A and C illustrate one representative set of FACS analysis for each molecule. In A, untreated cells are represented by the black trace, Ht31 treated cells are indicated by the red trace, and Ht31p treated cells are shown as the blue trace. In C, LPS treated cells are illustrated by the orange trace, LPS with Ht31 are red and LPS with Ht31p are blue. The graphs in columns B and D represent the average percent inhibition (B) or average percent increase (D) from 8 or 5 donors, respectively. The data illustrate that Ht31 inhibits the cell surface expression of CD18 by nearly 30% and CD11a by 10% (Figure 8 A and B). Expression of CD18 and CD11a in the presence of LPS was not significantly altered (Figure S1 A and B). Expression of CD83 was consistently increased in cells treated with Ht31 (Figure 8 A and B). In LPS treated cells, CD40, CD80 and CD83 expression increased appreciably as expected, but this increase was significantly inhibited by Ht31 and not Ht31p (Figure 8 C and D). Expression of CD11b was increased by Ht31 but not significantly, and no significant changes were found in CD11b expression in the presence of LPS (Figure S1 A–D). Expression of CD49d was significantly inhibited by Ht31, but the same level of inhibition was consistently detected in the presence of Ht31p (Figure S1 E and F). No significant changes were detected in the expression of CD11c, CD86 or HLADR in the presence of Ht31 or Ht31p with or without LPS (Figure S1 E and F). These data are consistent with the idea that PKA anchoring is required for proper cell surface expression of adhesion and costimulatory molecules on immature DC and during DC maturation.

**Figure 9:**
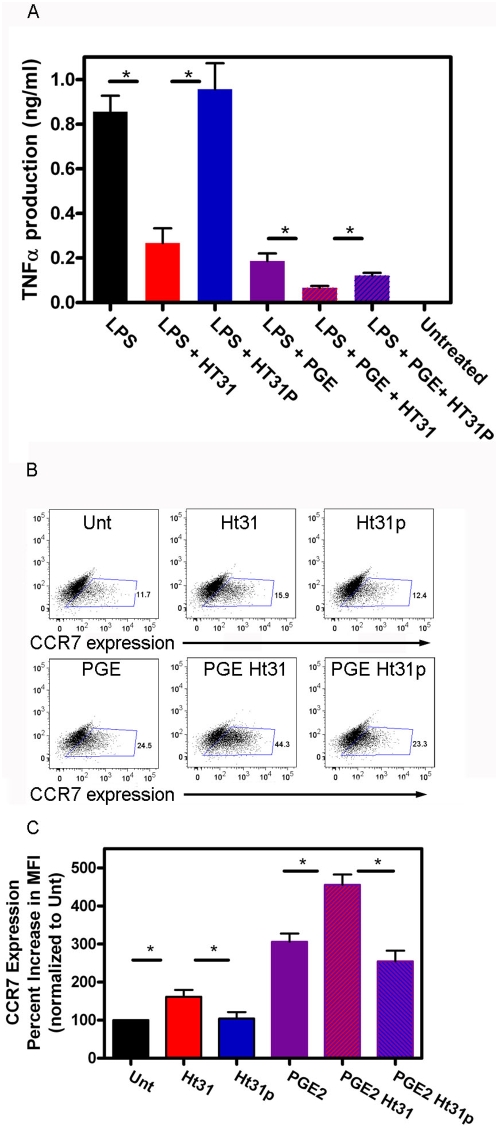
Ht31 inhibits TNF-α production and CCR7 expression. A) Day 5 DC were incubated with 10 ng/ml LPS without (Unt, black bars) or with 100 µM Ht31 (red bars) or Ht31p (blue bars) and 1 µM PGE_2_ without (purple bars) or with 100 µM Ht31 (purple and red hatched bars) or Ht31p (purple and blue hatched bars) for 24 hours at 37°C, 5% CO_2_. Supernatants were collected and used in TNF-α ELISA (Ebioscience). Three experiments were averaged. Error bars are S.E.M. Student TTEST reveals significance, P<0.05(*). B, C) Day 5 DC were incubated without (Unt, black bars) or with 100 µM Ht31 (red bars) or Ht31p (blue bars) and 1 µM PGE_2_ without (purple bars) or with 100 µM Ht31 (purple and red hatched bars) or Ht31p (purple and blue hatched bars) for 48 hours at 37°C, 5% CO_2_. Cells were collected, blocked, and stained for cell surface expression with anti-CCR7. FACS analysis was performed on a LSRII using FacsDiva software. B) One representative dot plot for each treatment. C) Three experiments normalized to untreated and averaged. Error bars are S.E.M. Student ttest reveals significance, P<0.05(*).

While the experiments presented to this point fit a model in which PKA activation stimulates DC and inhibiting PKA signaling by disrupting anchoring blocks this stimulatory signal, two sets of experiments resulted in data that does not fit with this model. TNF-α secretion was measured by ELISA from day 5 DC stimulated with LPS. In agreement with published studies, incubating cells with 1 µM PGE_2_ inhibits TNF- α production from DC an average of 82% (Figure 9A, purple bar). Interestingly, incubating DC with Ht31 (100 µM) also inhibited the secretion of TNF-α from LPS stimulated DC by an average of 74% (Figure 9A, red bars). The control peptide Ht31p has no significant effect on TNF-α secretion (Figure 9A, blue bars). Incubation with both Ht31 and PGE_2_ increase the average inhibition to nearly 95% (Figure 9A, red and purple hatched bars) while incubation with Ht31p again had no effect on the inhibition mediated by PGE_2_ (Figure 9A, blue and purple hatched bars). To confirm that the inhibition of TNF-α production was not on contaminating T cells instead of the DC, we matured DC from CD14^+^ negatively selected monocytes (98% pure as determined by FACS), stimulated with LPS in the presence or absence of Ht31 or Ht31p and assayed TNF-α secretion. The results obtained from monocyte derived DC were similar to those from adherence derived DC (data not shown). Furthermore, Ht31 had no effect on TNF-α secretion from unstimulated cells (data not shown).

In addition, the expression of the chemokine receptor CCR7 was enhanced in the presence of PGE_2_ and Ht31. Day 5 DC were incubated with 1 µM PGE_2_ in the absence or presence of 100 µM Ht31 or 100 µM Ht31p. Cells were collected, stained for CCR7 expression and analyzed by FACS. The data presented in figure 9b illustrate an increase of CCR7 expression in a population of cells. The numbers represent the % events in the boxed area. The average results from three independent donors were normalized to untreated and presented as percent increase in mean fluorescence intensity. Ht31 increases CCR7 expression by 60% (Figure 9c, red bars), PGE_2_ increases CCR7 expression by 200% (Figure 9c, purple bars), and PGE_2_ with Ht31 increases CCR7 expression by an additional 150% (350% increase over untreated) (Figure 9c, red and purple hatched bars). The Ht31p peptide does not increase CCR7 expression on its own (Figure 9c, blue bars) or with PGE_2_ (Figure 9c, purple and blue hatched bars). The effects of PGE_2_ on CCR7 expression have been correlated with increased migratory capacity of DC [2], suggesting that Ht31 may also be increasing the migratory capacity of DC.

## Discussion

The data presented here portray the first evidence for expression of AKAPs in dendritic cells and illustrate a significant role for anchored PKA in regulating several aspects of DC biology. Using western analyses we identified five AKAPs: AKAP149, AKAP95 and AKAP79, Ezrin, and AKAP-Lbc. We have also illustrated an increase in expression of AKAP149, AKAP79 and AKAP-Lbc with DC maturation. The anchoring inhibitor peptides Ht31, IS and RIAD were utilized to provide functional evidence for the importance of anchored PKA activity in DC. Ht31 peptide has been previously used to demonstrate the importance of PKA anchoring in T cells, cardiac cells and neuronal cells [11], [12], [16], [17]. IS and RIAD have been utilized to demonstrate isoform specific responses in neuronal and T cell signaling [14], [15]. Our data suggest that anchored type II PKA regulates the ability of a DC to present antigen and activate a T cell. Anchored PKA also regulates secretion of both pro-inflammatory and immunosuppressive cytokines, and regulates the cell surface expression of costimulatory molecules, integrins and the chemokine receptor CCR7.

Interpretation of these data requires some understanding of the multiple roles of PKA in regulating DC function and subsequent immune responses. PKA can be activated by cell permeable analogs of cAMP, by the adenylyl cyclase activator, forskolin, by prostaglandins and other G-protein coupled receptor agonists and by toxins [10]. Activation of PKA by cell permeable analogs of cAMP, forskolin, cholera toxin and lymphotoxin has been shown to activate DC, as characterized by increases in cell surface expression of CD80, CD83, CD86 and HLA-DR molecules, and the enhanced ability to present alloantigen in an MLR leading to increased T cell proliferation [7]. In addition, PGE_2_ has been shown to increase CCR7 expression and promote DC migration [5]. In contrast, much evidence also exists supporting the notion PKA inhibits DC function. LPS stimulated antigen presentation and T cell proliferation are also inhibited by PGE_2_ and cAMP analogs [3]. Inhibition of the immune response by activated DC is further supported by evidence that PGE_2_ and cAMP increase IL-10 secretion and decrease the ability to present antigen and stimulate T cell proliferation [18]. These effects have been summarized by Harizi and colleagues who state that “PGE_2_ has stimulatory and inhibitory effects on the activation of the DC depending on the site of encounter. In peripheral tissues, PGE_2_ seems to have a stimulatory effect of DC inducing their activation and migration [4], [5]. Once the cells have migrated to lymphoid organs, PGE_2_ assumes a suppressive action, inhibiting the maturation of DC and their ability to present antigen [19]” [18]. Of note, however, is that of the studies referred to above, those performed on DC of murine origin are inhibited by PKA activators, while DC of human origin are stimulated by PKA activators. The results presented here agree with those published using human DC that PKA activators stimulated antigen presentation, inhibited TNF production and stimulated CCR7 expression.

Historically, disruption of PKA anchoring with Ht31 has mimicked the effect of the PKA inhibitors such as PKI peptide [20], [21]. These results have been interpreted to mean that 1) PKA is being inhibited through delocalization and the subsequent inability to be activated by localized increases in cAMP, 2) signaling by PKA cannot be initiated due to delocalization from nearby substrates. In agreement with this model, PKA activators stimulated antigen presentation and the anchoring inhibitor Ht31 blocked the activation by PGE_2_ and forskolin. Ht31 and AKAP-IS also inhibited antigen presentation on their own. Interestingly, however, PGE_2_ and Ht31 both inhibited TNF-α production from LPS stimulated DC and both PGE_2_ and Ht31 increased CCR7 expression in maturing DC. The effects of PGE_2_ and Ht31 were additive, and the effects of neither PGE_2_ nor Ht31 were blocked by PKA inhibitors PKI or H89 (data not shown). While at first glance these data could suggest a PKA independent mechanism for PGE_2_ and Ht31, PKA mediated signaling events that cannot be blocked by H89 have been reported [22], [23], and the PKI peptide may be degraded in the multi-day assays presented here. Thus it is possible that these events are PKA mediated and that Ht31 is mimicking PGE_2_. Further experimentation is necessary to explore this hypothesis.

Cyclic AMP analogs and PGE increase IL-10 production in DC [24]. While PKA inhibitor studies have not been conducted in DC, data in macrophages illustrate that cAMP increases IL-10 production, and incubation with the PKA inhibitor H89 blocks the cAMP-mediated IL-10 induction [25]. From these data we suggest that Ht31 is inhibiting PKA signaling and regulating IL-10 secretion. The exact mechanism is not known, but one study in macrophages may suggest that cAMP regulates IL-10 secretion through posttranscriptional mechanisms [26]. Additional studies will have to be conducted to determine how Ht31 is inhibiting IL-10 secretion in DC.

Anchored PKA activity was found to be important in regulating the cell surface expression of co-stimulatory molecules CD40, CD80 and CD83. Similar to the PGE studies referenced above, Ht31 significantly increased expression of CD83. Furthermore, Ht31 significantly decreased the expression of CD40, CD80, and CD83 on LPS stimulated cells. Naïve T cells are dependent on costimulation to become fully active. While effector T cells are not dependent on costimulation, costimulation will lower the threshold for activation. Thus the decrease in expression of costimulatory molecules CD40, CD80, and CD83 may be increasing the threshold for activation causing a decrease in antigen presentation.

Experiments illustrating a slight but significant decrease in integrin expression may also be a lead as to the mechanism by which Ht31 is inhibiting antigen presentation. Integrins are noncovalently linked αβ heterodimers, with the specific αβ pairs linked to unique components of the cytoskeleton and downstream signaling pathways. Integrins regulate DC trafficking and immune synapse formation through interactions with intracellular adhesion molecules (ICAM) [27]–[29]. A significant body of literature has accumulated on the involvement of PKA signaling, integrins and cell motility [30]. Recent findings illustrate that the α4β1 integrin is an AKAP [31], that α4β1 and α5β1 integrin ligation activates PKA at the leading edge of cells [32], and that PKA regulates crosstalk between α4β1 and αvβ3 integrins [33]. The distribution of integrins on DC appears to be a developing field with much of the focus on the β2 family [27]. However, more recent studies illustrate that PGE_2_ and α5β1 integrins regulate DC migration [2]. While we could not find studies demonstrating a direct regulation of integrins by PKA in DC, several studies have demonstrated roles for regulation of β2 integrins by PKA in neutrophils. In neutrophils, PKA activation inhibits adhesion and promotes migration, while inhibition of PKA stimulates adhesion [34], [35]. PKA phosphorylates L-plastin (LPL), a leukocyte specific actin bundling protein that is associated with activation of αMβ2 integrin mediated adhesion [36]. These data support the idea that PKA is important in regulating integrin activity and actin polarization, and therefore may be important in regulating antigen presentation. Our studies on CD11a/CD18 (integrins αLβ2 or LFA), CD11b/CD18 (integrin αMβ2, Mac-I or CR3), and CD11c/CD18 (integrin αXβ2, CR4) illustrate that Ht31 significantly inhibits the cell surface expression of CD18 (β2 integrin), and slightly inhibits the expression of the CD11a (αL) and CD11b (αM) subunits. Further experimentation is necessary to determine if Ht31-mediated changes in integrin expression are responsible for inhibition of antigen presentation and if Ht31 is regulating integrin activity.

Ht31 has been shown to disrupt the PKA/AKAP interaction of nearly all AKAPs [37]. For each AKAP we have identified, possible mechanisms for regulating antigen presentation can be proposed. Since the most recent comprehensive AKAP review was published [38], several studies have been conducted that may be relevant to regulation of antigen presentation. As such we have briefly reviewed aspects of each AKAP identified here and how it may be implicated in regulating DC biology.

AKAP149 and AKAP95 both bind to the novel myc binding protein AMY-1 [39]. AMY-1 is a stimulating factor for differentiation in erythrocytes, and competes for PKAc binding to the AKAP. Given that AKAP95 and AKAP149 protein expression are regulated differentially in monocytes versus immature and mature DC, the ability of each AKAP to interact with AMY-1 may play a role in regulating DC maturation.

AKAP79 is associated with the β-adrenergic G-protein coupled receptor [40], [41]. This association facilitates phosphorylation of the β-adrenergic receptor by PKA and switches signaling from G_s_ coupled activation of adenylyl cyclase to G_i_ coupled activation of the MAP kinase pathway [42]. Phosphorylation of the β-adrenergic receptor by PKA also promotes recycling and resensitization of the receptor [43]. β_2_-agonists inhibit IL-12 production from DC stimulated with CD40-CD40L interaction [44], suggesting that β-adrenergic receptors are present in DC and may be regulated by AKAP79.

In addition to binding PKA, AKAP-Lbc is a Rho-guanine nucleotide exchange factor (GEF) [45], [46]. AKAP-Lbc Rho-GEF activity is activated via adrenergic signaling and Gα12 and inactivated through interactions with 14-3-3 [47]. Activation of Rho has been shown to regulate actin polarization and the formation of immune synapses in DC [48]. Hence the presence of AKAP-Lbc in DC may indicate a mechanism for regulating antigen presentation by way of influencing immune synapse formation.

Ezrin, a member of the ezrin-radixin-moesin (ERM) family of cytoplasmic linker proteins, has been shown to regulate T cell activation via anchoring of PKA to lipid rafts [12]. Ezrin also associates with clustered ICAMs and becomes phosphorylated on serine, threonine, and tyrosine residues [49]. Precedence exists for an ERM domain containing protein, talin, to activate integrin signaling and clustering [50]. Thus it will be interesting to determine if anchoring of PKA to Ezrin might be regulating antigen presentation through integrin activation.

In this study we looked at the role of PKA anchoring in regulating DC biology. Using the Ht31 inhibitor and Ht31p control peptides in DC loaded with protein or peptide antigen, we illustrate that type II PKA anchoring is necessary for optimal antigen presentation and activation of antigen specific T cells and activation of effector T cells in an allo response. Disruption of PKA anchoring inhibits TNF-α and IL-10 production and regulates the expression of co-stimulatory molecules, integrins, and the chemokine receptor, CCR7. Thus, the presence and functional importance of AKAPs in DC open an exciting new avenue of research in understanding the molecular mechanisms of antigen presentation, T cell activation and DC biology.

## Materials and Methods

### Cells

Human subjects protocols and consent forms were approved by the Oregon Health & Science University Institutional Review Board (IRB). Source leukocytes purchased from the Portland Red Cross had Red Cross IRB approval and thus were exempt from VA and OHSU IRB. Leukocytes obtained in house were obtained with written consent from donors. Blood was obtained from the Red Cross immediately after being drawn and processed to isolate peripheral blood mononuclear cells (PBMC). 45–50 mls of source leukocytes were diluted 1∶1 with sterile PBS and layered on a Ficoll gradient (25 mls of diluted blood: 12.5 mls Ficoll Paque-Plus (Amersham-Pharmacia). The gradient was centrifuged for 30 minutes at 1100×g at room temperature with the brake turned off. The interface was collected and washed two times with RPMI 1640 (Invitrogen-Gibco-BRL). Cells were pelleted by centrifugation at 250×g for 10 minutes, resuspended in freezing media (25% FCS, 12% DMSO, RPMI 1640) at 25–50 million per vial, frozen at −80°C in Mr. Frosties (Nalgene) and transferred to liquid nitrogen for long term storage. These cells represent PBMCs. Monocytes, CD14^+^ cells, (day 0) were obtained from PBMCs by negative selection using either EasySep human monocyte enrichment kit (Stem cell technologies) or Dynal monocyte negative selection kit (Invitrogen) per manufacturer's protocol. Cells were analyzed by flow cytometry and found to be >95% CD14^+^. Immature dendritic cells (day 5 DC) were made by culture of purified monocytes with 10 ng/ml GM-CSF and 10 ng/ml IL-4 in RPMI 1640 with 10% human serum (a generous gift of Drs. David and Deborah Lewinsohn) for 5 days. Mature dendritic cells (day 7 DC) were made by culturing day 5 DC with 10 ng/ml GM-CSF, 10 ng/ml IL-4, and 10 ng/ml LPS in RPMI 1640 with 10% human serum for additional two days.

### Western analysis

Day 5 and Day 7 DC were collected as follows: 1) non-adherent cells in media were transferred to a conical tube at 4°C, 2) the remaining adherent cells were incubated in cell dissociation media (CDM, Sigma, St. Louis, MO) at 37°C for 15 min, 3) the flask was whacked vigorously and the cells were added to the conical tube in (1), 4) the flask was rinsed with PBS and added to (1), 5) the cells were pelleted by centrifugation at 800×g for 15 min at 4°C. The pellet was rinsed with PBS and lysed with boiling Laemmli sample buffer for 5 minutes. Protein concentration was determined using SPN-Protein assays (G-Biosciences, St. Louis, MO). Samples (10 µg total protein) were subjected to SDS-PAGE and transferred to PVDF membrane (Immobilon-P, Millipore). After transfer, membranes were blocked in 5% milk TTBS for 1 hr. Primary antibodies were incubated for 1 hr at RT in TTBS at the following dilutions: Ezrin (1∶1000) (Thermoscientific, Waltham, MA), GAPDH (1∶1000) (Imgenex, San Diego, CA) AKAP79 (1∶250), AKAP149 (1∶250), AKAP95 (1∶100), RI (1∶500) and RII (1∶1000) (BD Biosciences, San Jose, CA). Secondary antibodies, goat anti-rabbit and goat anti-mouse horseradish peroxidase conjugates, were used at 1∶5000 for 1 hr at RT (Santa Cruz Biotechnology). NEN Renaissance chemiluminescence was used for detection.

### AKAP-Lbc western

Frozen day 5 DC (2–3×10^6^) were lysed in 50 mM Tris-HCl pH 7.5, 0.2% deoxycholate, 1% Triton-X, 150 mM NaCl for 30 minutes on ice and spun at 10,000×g at 4°C for 10 minutes. Laemmli sample buffer was added to the supernatant and samples were boiled for 5 minutes at 97°C. Protein concentration was determined using SPN-Protein assays (G-Biosciences, St. Louis, MO). Samples were run on large 7% SDS-PAGE gels overnight and transferred to Immobilon-P membrane. Membranes were blocked in 5% milk-TTBS for 1 hour, incubated with anti-AKAP-Lbc antibody (generously provided by Dr. Dario Diviani) at 1∶1000 dilution, and goat-anti-rabbit HRP conjugated secondary antibody at 1∶5000 dilution (Santa Cruz biotechnology). NEN Renaissance chemiluminescence was used for detection.

### Immunofluorescence

Day 5 DC were generated by plating day 5 DC onto poly-l-lysine (Sigma-Aldrich) coated round coverslips in a 12 well plate and incubating in RPMI 10% human serum for 3 hours. Day 7 DC were generated by plating day 5 DC onto poly-l-lysine (Sigma-Aldrich) coated round coverslips in a 12 well plate and incubating in RPMI 10% human serum with GM-CSF, IL-4, and LPS (10 ng/ml final concentrations) for two days. The DC were fixed with 2% paraformaldehyde in PBS for 1 hr, washed with PBS and blocked with 2% human serum, 2% goat serum and 0.5% fetal calf serum in PBS with 0.1% saponin 15 minutes to overnight at 4°C. DC were washed 3×with 1 ml of PBS+0.1% saponin and incubated with primary antibody (1∶100 dilution) in PBS+0.1% saponin in a moist staining chamber for 1 hr. The primary antibodies are the same as those used for westerns with the exception of RIIβ (anti-rabbit antibody, Biomol, PA), and FITC conjugated HLA-DR (BDbiosciences). DC were again washed 3×with 1 ml of PBS+0.1% saponin and incubated with secondary antibody (donkey anti mouse or donkey anti rabbit, FITC or TxRd, Jackson Immuno Research Laboratories, West Grove, PA) in PBS+0.1% saponin in a moist staining chamber for 1 hr. Hoescht stain (20 ng/ml final) was added with the secondary antibody where indicated. Finally, DC were washed two times with PBS and one time with milliQ H20, and then mounted using Fluoromount G (Southern Biotech, Birmingham, AL).

### IFN-γ detection by ELISPOT

Antigen specific activation of T cells was detected by measuring interferon-γ (IFN-γ) production by ELISPOT. 96-well nitrocellulose-backed plates (MAHA S4510; Millipore, Bedford, MA) were coated as recommended by the manufacturer with 10 µg/ml capture mouse anti-IFN-γ in 0.1 M NAHCO_3_ pH 9.6 (1-D1K; Mabtech, Nacka, Sweden) overnight at room temperature. Plates were then washed 3×15 min. with PBS, and blocked with RPMI/10% human serum for 1 h at room temperature. Day 5 DC (1–2×10^4^/well) were loaded with the indicated concentration of CFP10 peptide or protein antigen for 1 hr at 37°C, 5% CO_2_. CD4^+^ clonal T cells specific for CFP10 were then added (2×10^4^/well), and the plate was incubated overnight at 37°C, 5% CO_2_. Addition of 100 µM Ht31 or Ht31p (Promega, Madison, WI) or the indicated concentrations of IS, SCR, RIAD, RSCR (IS; AMAQIEYLAKQIVDNAIQQAKG-RRRRRRRRRRR, SCR; AMAQDVEIQLKAAYNQKLIAIG-RRRRRRRRRRR, RIAD; LEQYANQLADQIIKEATEK-RRRRRRRRRRR, RSCR; IEKELAQQYQNADAITLEK-RRRRRRRRRRR (Biomatik, Cambridge, Ontario, Canada)) occurs 20 minutes before addition of antigen. After washing six times with PBS/0.05% Tween 20 (Sigma), 100 µl of 1 µg/ml biotinylated secondary anti-IFN-γ mAb (7B6-1; Mabtech) in 0.5%FBS in PBS was added and incubated for 2 h at room temperature. Plates were then washed again 6×with PBS/0.05% Tween 20, 100 µl avidin/biotinylated enzyme (HRP) complex (Vectastain ABC Elite Kit; Vector, Burlingame, CA) was added to wells, and the plates were incubated for 1 h. Plates were again washed 6×with PBS/0.05% Tween 20, then 100 µl of 3-amino-9-ethylcarbazole substrate (Vectastain AEC substrate kit; Vector) was added. After 4–10 min, the colorimetric reaction was stopped by washing thoroughly with distilled water, and plates were air-dried. Spots were quantitated using an AID ELISPOT High Resolution Reader System (Cell Technology, Inc, Columbia, MD).

### Mixed Lymphocyte Reaction

Ten thousand DC matured from CD14^+^ monocytes (Easy Sep) with GM-CSF and IL-4 (see above) were plated in 96 well nitrocellulose-backed plates with 125,000 or 62,500 PBMC for three days at 37°C, 5% CO_2_. IFN-γ was detected by ELISPOT as described above.

### ELISA

In a 96 well plate, 15–30,000 adherence isolated day 5 DC were incubated with 10 ng/ml LPS without or with 100 µM Ht31 or Ht31p for 16–18 hrs. Supernatants were collected and 100 µl was used in an IL-10 ELISA (R&D Systems) as per manufacturer protocol. For TNF-α ELISA, CD14^+^ monocytes were isolated by negative selection (as described above) and incubated for 5 days with GM-CSF and IL-4 (both 10 ng/ml). These day 5 DC were plated in a 96 well plate at 5,000/well and incubated without or with LPS (10 ng/ml) and without or with Ht31 (100 µM) or Ht31p (100 µM) and PGE_2_ (1 µM), where indicated, for 24 hrs at 37°C, 5% CO_2_. Supernatants were used in a TNF-α ELISA as per manufacturers protocol (Ebioscience).

### ANNEXIN V/PI STAINING

Adherence isolated day 5 DC were collected, plated at 200,000 per well in a 96 well ULA plate and incubated without or with 100 µM Ht31 or Ht31p for 18 hours at 37°C, 5% CO_2_. Cells were collected, rinsed with PBS and stained with annexin V and propidium iodide (PI) per manufacturers protocol (BD Pharmingen).

### FACS

Adherence isolated day 5 DC were collected, plated at 100,000 per well in a 96 well ULA plate and incubated without or with 10 ng/ml LPS or 1 µM PGE_2_, without or with 100 µM Ht31 or Ht31p for 48 hrs at 37°C, 5% CO_2_. Cells were collected rinsed with PBS, blocked with FACS buffer (2% human serum, 2% goat serum, 0.5% fetal bovine serum in PBS) for 30 minutes at room temperature and stained with conjugated antibody for 45 minutes at 4°C, FITC-conjugated anti-CD18, PE-conjugated anti-CD49, FITC-conjugated anti-CD11a, PE-conjugated anti-CD11b, PE-conjugated anti-CCR7, APC-conjugated anti-CD11c (Ebioscience) and FITC-conjugated anti-CD80, PE-conjugated anti-CD40, PE-conjugated anti-CD83, APC-conjugated anti-CD86, FITC-conjugated anti-HLA-DR (BDbiosciences). Acquisition was performed with an LSRII flow cytometer using FACS Diva software. All analyses were performed using FlowJo software (TreeStar).

## Supporting Information

Figure S1 Day 5 DC were incubated without or with 10 ng/ml LPS and 100 µM Ht31 or Ht31p for 48 hours. Cells were collected, blocked, and stained for cell surface expression using conjugated antibodies, see materials and methods. FACS analysis was performed on a LSRII using FacsDiva software. A, E upper panels) One representative histogram showing untreated cells (black trace), Ht31 treated cells (red trace), and Ht31p treated cells (blue trace). B) Graphical representation of percent inhibition of mean fluorescent intensities compared to untreated samples, data from eight donors was normalized and averaged. C, E lower panels) One representative histogram showing untreated cells (black trace), LPS treated cells (orange trace), LPS+Ht31 (red trace), and LPS+Ht31p (blue trace). D) Graphical representation of percent increase in mean fluorescent intensity compared to untreated cells, data from five donors was normalized and averaged. F) Graphical representation of average mean fluorescent intensity (MFI), data from at least three donors was normalized and averaged.
(1.01 MB TIF)
