# Supplementary figures and images for: A-Kinase Anchoring in Dendritic Cells Is Required for Antigen Presentation
Source: PLoS One. 2009 Mar 11;4(3):e4807. doi: 10.1371/journal.pone.0004807 (PMC2652104; doi:10.1371/journal.pone.0004807)

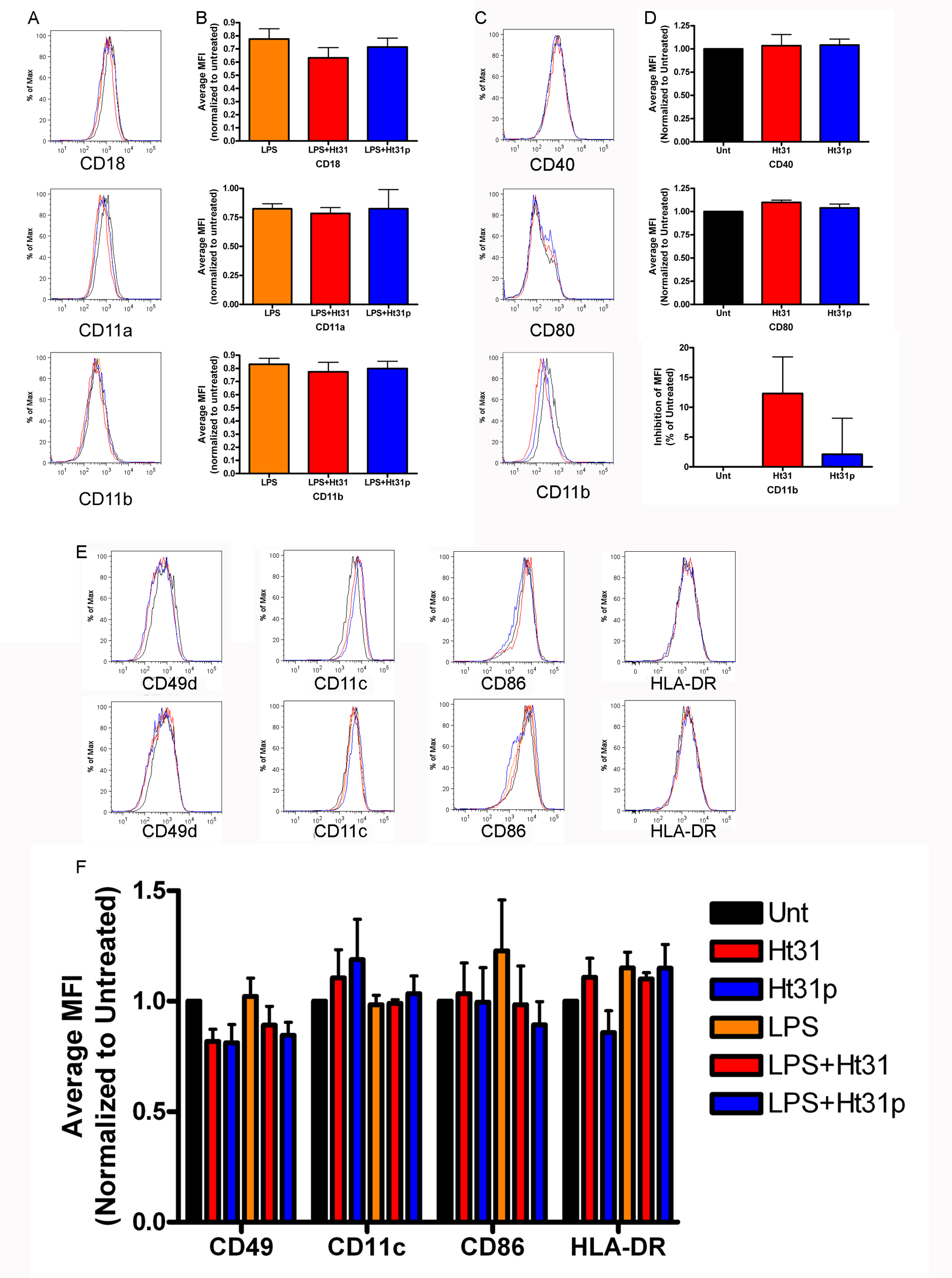

Supplement: Figure S1 — Day 5 DC were incubated without or with 10 ng/ml LPS and 100 µM Ht31 or Ht31p for 48 hours. Cells were collected, blocked, and stained for cell surface expression using conjugated antibodies, see materials and methods. FACS analysis was performed on a LSRII using FacsDiva software. A, E upper panels) One representative histogram showing untreated cells (black trace), Ht31 treated cells (red trace), and Ht31p treated cells (blue trace). B) Graphical representation of percent inhibition of mean fluorescent intensities compared to untreated samples, data from eight donors was normalized and averaged. C, E lower panels) One representative histogram showing untreated cells (black trace), LPS treated cells (orange trace), LPS+Ht31 (red trace), and LPS+Ht31p (blue trace). D) Graphical representation of percent increase in mean fluorescent intensity compared to untreated cells, data from five donors was normalized and averaged. F) Graphical representation of average mean fluorescent intensity (MFI), data from at least three donors was normalized and averaged. (1.01 MB TIF) [file pone.0004807.s001.tif]
